# Polyquaternium-mediated delivery of morpholino oligonucleotides for exon-skipping *in vitro* and in *mdx* mice

**DOI:** 10.1080/10717544.2017.1337827

**Published:** 2017-06-20

**Authors:** Mingxing Wang, Bo Wu, Sapana N Shah, Peijuan Lu, Qilong Lu

**Affiliations:** McColl-Lockwood Laboratory for Muscular Dystrophy Research, Cannon Research Center, Carolinas Medical Center, Charlotte, NC, United States

**Keywords:** Polyquaternium, antisense delivery, exon-skipping, PMO, muscular dystrophy

## Abstract

Antisense oligonucleotide therapy for Duchenne muscular dystrophy has shown great potential in preclinical and clinical trials, but its therapeutic applications are still limited due to inefficient delivery. In this study, we investigated a few polyquaterniums (PQs) with different size and composition for their potential to improve delivery performance of an antisense phosphorodiamidate morpholino oligomer (PMO) both *in vitro* and *in vivo*. The results showed that Luviquat^TM^ series, especially PQ-1 and PQ-3, promoted the exon-skipping efficiency comparable to Endoporter-mediated PMO delivery *in vitro*. Significant enhancement in skipping dystrophin exon 23 has also been achieved with PQ-3 up to seven-fold when compared to PMO alone in *mdx* mice. Cytotoxicity of the PQs was lower than Endoporter and PEI 25 K *in vitro* and muscle damage not clearly detected *in vivo* under the tested concentrations. These results together demonstrate that the optimization of PQ in molecular size, composition and distribution of positive charges is the key factor to achieve enhanced PMO exon-skipping efficiency. The higher efficiency and lower toxicity endow polyquaternium series as AO delivery enhancing agents for treating muscular dystrophy and other diseases.

## 1. Introduction

Antisense therapy is a powerful technology for the treatment of genetic disorders or infections. Among them, antisense oligonucleotide (AO)-mediated exon-skipping has been demonstrated as a promising therapy to treat Duchenne muscular dystrophy (DMD) by facilitating ‘skipping’ of specific dystrophin gene exon(s) to restore the reading frame of the mutated transcripts (Hoffman et al., 1987; Koenig et al., 1989; Amantana et al., 2007; van Deutekom et al., 2007; Wu et al., 2008, 2009, 2010, 2012; Yin et al., 2008; Kinali et al., 2009; Goemans et al., 2011; Mendell et al., 2013). AOs are short, single-stranded sequences of synthetic pieces of chemically modified RNA or DNA, which have the ability to hybridize to specific targets by the Watson–Crick base-pairing rules, and are easier to scale up for GMP production than for viral vectors or cell therapies (Evers et al., 2015). Of the synthetic oligonucleotide chemistries, phosphorodiamidate morpholino oligomer (PMO) is the most widely used structure for exon-skipping in the dystrophin gene and has been approved by FDA as the only drug specific to DMD (Fletcher et al., 2006; Cirak et al., 2011; Goemans et al., 2011; Malerba et al., 2011; Mendell et al., 2013). PMO, as a synthetic mimic of nucleic acid, has replaced the deoxyribose rings with morpholino rings linked through phosphorodiamidate intersubunits, being neutral under physiological condition and exhibiting excellent stability and lower toxicity compared with other oligonucleotide chemistries (Summerton & Weller, 1997; Yano & Smyth, 2012). However, the uncharged nature of PMOs is associated with poor cell uptake and fast clearance in bloodstream, which dramatically impede pharmacological outcomes. Studies in several animal models have demonstrated that a significant therapeutic effect can only be achieved with high doses of PMOs, which is cost inhibitive and potential long-term risk of toxicity (Wu et al., 2010). To improve delivery efficiency, chemically modified PMOs with cell-penetrating peptides or dendrimeric octa-guanidines have been reported with significant heightening in targeting skipping of dystrophin exons, leading to near normal levels of dystrophin expression in muscles throughout the body by systemic delivery (Amantana et al., 2007; Wu et al., 2008, 2009, 2012; Yin et al., 2008). However, the packed cationic modification is associated with higher toxicity, with LD50 only near 10 mg/kg, making it unsuitable for clinical applications (Wu et al., 2008, 2012). Furthermore, the complicated synthesis and purification in modification make it more expensive, and possible peptide-related immune responses might prevent repeated administration. Recently, some small molecules enhanced exon-skipping of AOs have been also reported, such as dantrolene-aided PMO delivery reported by Kendall et al. (2012), and monosaccharide-formulated AOs reported by Yin’s group (Cao et al., 2016; Han et al., 2016) which have been demonstrated promoted uptake and activity of AOs in *mdx* mice. The polymer-vectored gene/AO delivery has attracted much attention, because of their structural flexibility chosen from synthetic or natural compounds, larger capacity for therapeutic agent, ease in handling and less cost than viral vector. Polymer-based oligonucleotide delivery can lower the oligonucleotide dose required for desired pharmacological outcomes and thus can further reduce the potential of toxicity caused by the therapeutic oligonucleotides.

Previously, we reported a few cationic polyelectrolyte (PE)-mediated PMO delivery, found that polydiallyldimethylammonium chloride (PDDAC) with high molecular size improved the delivery efficiency of PMO significantly *in vitro* and *in vivo*. However, the higher positive charges of PDDAC narrow the dosage window *in vivo* (Wang et al., 2015). The results demonstrated that the optimization of polymer in composition [structure, molecular size, hydrophilic–lipophilic balance (HLB) and charges] is the crucial factor to achieve enhanced PMO exon-skipping efficiency. Considering these factors, we investigated here several polyquaterniums (PQs) as delivery carriers for antisense PMO *in vitro* and in *mdx* mice. PQ is a kind of the polycations with quaternary ammonium centers and has been explored for biochemical and medical applications, such as being applied in cosmetic ingredients, hair condition or shampoo (Reich et al., 2005; http://dewolfchem.com/wp-content/uploads/2013/08/Luviquat-TDS.pdf). The permanent cationic characteristics such as valence, charge and structure would be expected to have an important impact on the oligonucleotide compaction and subsequent transfection. We therefore chose a few PQs with similar composition, but differ in charges and Mw for PMO delivery, with the aim to identify optimal HLB and reduce toxicity. The outcome demonstrated that the PQ-mediated PMO significantly enhanced exon-skipping compared with PMO alone both *in vitro* and *in vivo*, and reduced toxicity as compared to previously studied PE series. The results further indicate that the vector efficacy depends on the composition and charge distribution of molecules, which confirms the corresponding challenge to find and optimize vector for therapeutic agent delivery.

## 2. Materials and methods

### 2.1. Materials

**Figure 1.**
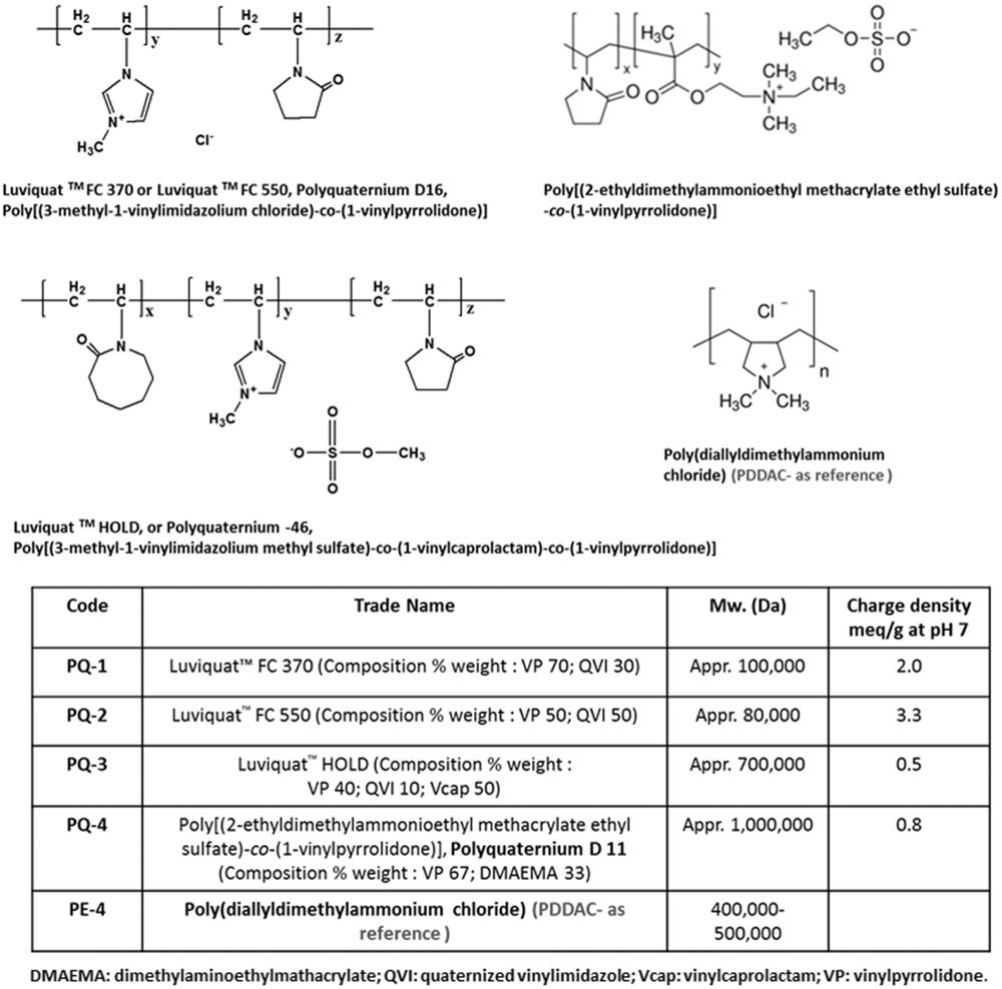
Polyquaternium (PQ) chemical structure, trade name and code.

Dulbecco’s modified Eagle’s medium (DMEM), penicillin–streptomycin, fetal bovine serum (FBS), L-glutamine and HEPES [4-(2-hydroxyethyl)-1-piperazineethanesulfonic acid] buffer solution (1 M) were purchased from GIBCO (Invitrogen Crop, Carlsbad, CA). Phosphorodiamidate morpholino oligomer PMOE50 (5′-AACTTCCTCTTTAACAGAAAAGCATAC-3′), PMOE23 (5′-GGCCAAACCTCGGCTTACCTGAAAT-3′) and Endoporter were purchased from Gene Tools (Philomath, OR). Polyquaterniums (PQs) and all other chemicals were purchased from Sigma-Aldrich (St. Louis, MO), unless otherwise stated. PQs applied in this study are commercially available and have been widely applied in the field of environment and biotechnology. Their structures and code names were illustrated in Figure 1.

### 2.2. Cell viability assay

Cytotoxicity was evaluated in C2C12E50 cell line using the MTS [3-(4,5-dimethylthiazol-2-yl)-5-(3-carboxymethoxyphenyl)-2-(4-sulfophenyl)-2H-tetrazolium]-based assay. Cells were seeded in a 96-well tissue culture plate at 1 × 10^4^ cell/well in 200 μL 10% FBS-DMEM. Cells achieving 70–80% confluence were exposed to polymer at different doses for 24 hours, followed by addition of 20 μL of Cell Titer 96^®^Aqueous One Solution Proliferation Kit (Promega Corporation, Madison, MI). After further four-hour incubation, the absorbance was measured at 570 nm using a Tecan 500 Plate reader (Tecan US, Inc, Morrisville, NC) to obtain the metabolic activity of the cell. Untreated cells were taken as controls with 100% viability and wells without cells as blanks, the relative cell viability was calculated by: (***A***_treated_−***A***_background_) × 100/(***A***_control_−***A***_background_). All viability assay was carried out in triplicate.

### 2.3. *In vitro* transfection

The C2C12E50 myoblast and C2C12E23 differentiated cell lines expressing the reporter GFP were used in this study. The expression of GFP was controlled by the effective skipping of the inserted human dystrophin exon 50 sequence (hDysE50) and mouse dystrophin exon 23 sequence (mDysE23), respectively (Hu et al., 2010).

#### 2.3.1. C2C12E50

The C2C12E50 cell line was maintained in 10% FBS-DMEM in a humidified 10% CO_2_ incubator at 37 °C. About 5 × 10^4^ cells/well in 500 μL 10% FBS-DMEM medium were seeded and allowed to grow until a confluence of 70%. Cell culture medium was replaced before addition of polymer/PMOE50 (PMO fixed at 5 μg, PEI 25 K and Endoporter used as comparison) formulation with varying ratio. All polymer/PMO complexes were prepared immediately before using by vertexing a mixture of PMO and polymer stock solution gently at designated polymer to PMO weight–weight (wt/wt) ratios. The final volume was adjusted to 50 μL and incubated 30 minutes at room temperature to allow self-assembly, and then premixed complexes were added to cells. Transfection efficiencies indicated by GFP production were recorded after three-day incubation with the Olympus IX71 fluorescent microscope (Olympus America Inc., Melville, NY) and digital images taken with the DP Controller and DP Manager software (Olympus America Inc., Melville, NY). Transfection efficiency was also examined quantitatively using flow cytometry. Cells were washed twice with phosphate**-**buffered solutions (PBS) (1×, pH 7.4), treated with 0.2 mL 0.05% trypsin–EDTA, followed by incubation for 3 minutes at 37 °C. The cells were then treated with cooled growth medium (1 mL), collected by centrifugation and then resuspended in 0.5 mL of ice-cold PBS (1X, pH 7.4). Samples were immediately run on a FACSCalibur flow cytometer (BD, Franklin Lakes, NJ). At least 1 × 10^4^ cells were counted and analyzed with CellQuest Pro (BD, Franklin Lakes, NJ) software package.

#### 2.3.2. C2C12E23

The cell culture and delivery protocol are the same as in C2C12E50, the images taken and cells collected after six-day treatment. Collected cells were initially washed twice with PBS, and RNA was extracted with TRIzol reagent (Invitrogen, Carlsbad, CA) as per manufacturer’s instructions. RNA was stored at −80 °C for later use. Reverse transcription polymerase chain reaction **(**RT-PCR) was performed with RT-PCR Master Mix (USB, Cleveland, OH) to amplify the sequence of interest. 100 ng of template RNA was used for each 25 μL RT-PCR. The primer sequences for the RT-PCR were eGFP5′, 5′-CAGAATTCTGCCAATTGCTGAG-3′ and eGFP3′, 5′-TTCTTCAGCTTGTGTCATCC-3′. The cycle conditions for reverse transcription were 43 °C for 15 minutes and 94 °C for 2 minutes. The reaction was then cycled 30 times at 94 °C for 30 seconds, 65 °C for 30 seconds and 68 °C for 1 minute. The products were examined by electrophoresis on a 1.5% agarose gel. The intensity of the bands was measured with the National Institute of Health (NIH) ImageJ 1.42 and percentage of exon-skipping was calculated with the intensity of the two bands representing both exon 23 unskipped and skipped as 100%. Unskipped band included exon 23 is 424 bp, and the exon 23 skipped band is 211 bp.

### 2.4. *In vivo* delivery

This study was carried out in strict accordance with the recommendations in Guide for the Care and Use of Laboratory Animals of the National Institutes of Health. The protocols were approved by the Institutional Animal Care and Use Committee (IACUC), Carolinas Medical Center (Breeding protocol: 10-13-07 A; Experimental protocol: 10-13-08 A). All injection was performed under isoflurane anesthesia, and all efforts were made to minimize suffering (Wu et al., 2008, 2009, 2010; Hu et al., 2010).

#### 2.4.1. Animals and injections

Dystropic *mdx* mice aged 4–5 weeks were used for *in vivo* testing (five mice each in the test and control groups) unless otherwise stated. The PMOE23 (5′-GGCCAAACCTCGGCTTACCTGAAAT-3′) targeting the boundary sequences of exon and intron 23 of mouse dystrophin gene (Gene Tools, Philomath, OR) was used. For intramuscular (i.m.) injections, 2 μg PMOE23 with or without polymer was formulated in 40 μL saline for each tibialis anterior (TA) muscle. For intravenous (i.v.) injection, 1 mg PMO with or without polymer (0.5 mg) in 100 μL saline was used. The muscles were examined 2 weeks later, snap-frozen in liquid nitrogen-cooled isopentane and stored at −80 °C.

#### 2.4.2. Immunohistochemistry and histology

Serial sections of 6 μm were cut from the treated mice muscles. The sections were stained with a rabbit polyclonal antibody P7 for the detection of dystrophin protein as described previously (Wu et al., 2008, 2010, 2012). Polyclonal antibodies were detected by goat anti-rabbit IgG Alexa 594 (Invitrogen, Carlsbad, CA). As for dystrophin-positive fiber counting, the number of dystrophin-positive fibers in one section was addressed using the Olympus BX51 fluorescent microscope (Olympus America Inc., Melville, NY).

#### 2.4.3. Western blot and RT-PCR for *in vivo* samples

Protein extraction and Western blot were done as described previously (Wu et al., 2008, 2010, 2012). The collected sections were ground into powder and lysed with 200 μL protein extraction buffer (1% Triton X-100, 50 mM Tris pH 8.0, 150 mM NaCl, 0.1% SDS), boiled at 100 °C water for 1 minute, then centrifuged at 18,000 × g for 15 minutes at 4 °C. The supernatants were quantified for the protein concentration with a protein assay kit (Bio-Rad Laboratories, Hercules, CA). Proteins were loaded onto a 4% to 15% Tris–HCL gradient gel. Samples were electrophoresed 4 hours at 120 V at room temperature. Then the gel blotted onto nitrocellulose membrane for 4 hours at 150 V at 4 °C. The membrane was probed with NCL-DYS1 monoclonal antibody against dystrophin rod domain (1:200 dilutions, Vector Laboratories, Burlingame, CA). The bound primary antibody was detected by HRP-conjugated goat anti-mouse IgG (1:3000 dilutions, Santa Cruz Biotechnology, Santa Cruz, CA) and the ECL Western Blotting Analysis System (Perkin-Elmer, Waltham, MA). The intensity of the bands of products obtained from the treated *mdx* mice muscles was measured and is compared with that of normal muscles of C57BL6 mice as 100% (National Institutes of Health, ImageJ software 1.42). α-Actin was detected by rabbit anti-actin antibody (Sigma, St. Louis, MO) as a sample loading control.

Total RNA was extracted from the muscle after dissection, and 100 ng of RNA template was used for a 25 μL RT-PCR with the Fidelitaq RT-MasterMix (USB, Cleveland, OH). The primer sequences for the RT-PCR were Ex20Fo 5′-CAGAATTCTGCCAATTGCTGAG-3′ and Ex26Ro 5′-TTCTTCAGCTTGTGTCATCC-3′ for amplification of mRNA from exons 20 to 26. The cycle conditions for reverse transcription were 43 °C for 15 minutes and 94 °C for 2 minutes. The reaction was then cycled 30 times at 94 °C for 30 seconds, 65 °C for 30 seconds and 68 °C for 1 minute. The products were examined by electrophoresis on a 2% agarose gel. Unskipped band included exon 23 is 1093 bp, and skipped band without exon 23 is 880 bp.

### 2.5. Measurement of serum creatine kinase and other components

Mouse blood was taken immediately after cervical dislocation and centrifuged at 1500 r.p.m. for 10 minutes. Serum was separated and stored at −80 °C. The level of serum components was determined by IDEXX Laboratories (North Grafton, MA).

### 2.6. Transmission electron microscopy

The polymer/PMO polyplex solution containing 1 μg of PMO was prepared at a weight ratio of 10/1 (PQ/PMO) in 100 μL 0.9% saline and analyzed using transmission electron microscopy (TEM; Philips CM-10; Philips Electronic North America Corp., Andover, MA). The samples were prepared using negative staining with 1% phosphotungstic acid. Briefly, one drop of sample solution was placed on a formvar and carbon-coated carbon grid (Electron Microscopy Sciences, Hatfield, PA) for 1 hour, and blotted dry, followed by staining for 3 minutes. Samples were analyzed at 60 kV. Digital images were captured with a digital camera system from 4 pi Analysis (Durham, NC).

### 2.7. Statistical analysis

All the results were expressed as mean ± SEM, and the data were analyzed using two-tailed Student’s *t*-test with a value of *p ≤ .05 being considered statistically significant.

## 3. Results and discussion

### 3.1. Cytotoxicity

**Figure 2.**
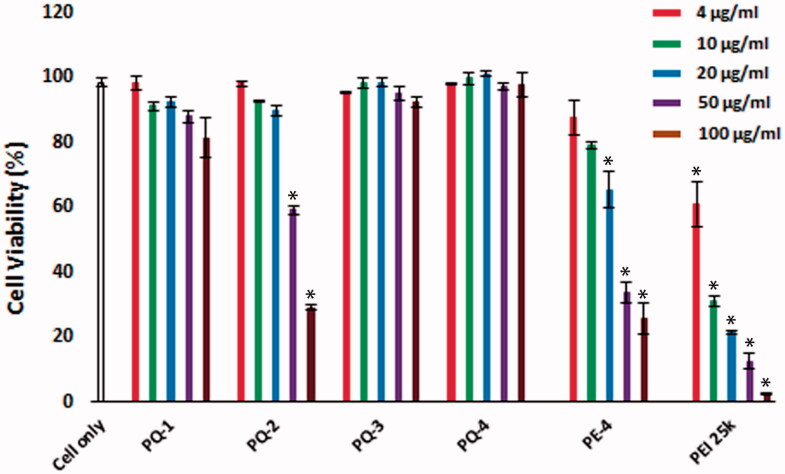
Cell viability of C2C12E50 myoblasts after treatment with PQs at 5 doses (4, 10, 20, 50 and 100 μg/mL for each polymer; PEI 25k is used as control) determined by MTS assay. Cells were seeded in 96-well plate at an initial density of 1 × 10^4^ cells/well in 0.2 mL growth media. The results are presented as the mean ± SEM (*n* = 3, two-tailed *t*-test, *p ≤ .05 compared with untreated cell).

The cytotoxicity of the PQs was determined using the MTS [3-(4,5-dimethylthiazol-2-yl)-5-(3-carboxymethoxyphenyl)-2-(4-sulfophenyl)-2H-tetrazolium]-based assay in C2C12E50 myoblast cell as shown in Figure 2. Toxicity of Luviquat^TM^ series including PQ-1, PQ-2 and PQ-3 was clearly charge-dependent, being higher with increasing QVI composition (cationic ingredient) in molecular structure of Luviquat^TM^. PQ-2 (Luviquat™ FC 550, composition % weight: VP 50; QVI 50, Mw ∼80,000; charge density: 3.0) showed higher toxicity than PQ-1 (Luviquat™ FC 370, composition % weight: VP 70; QVI 30, Mw ∼100,000; charge density: 2.0) or PQ-3 (Luviquat™ HOLD, composition % weight: VP 40; QVI 10 and Vcap 50, Mw ∼700,000; charge density: 0.5), although the PQ-3 is more hydrophobic and has the highest Mw. This result indicates that the toxicity is primary related to the % of QVI, but less to the VP segments. This has been further supported by low toxicity of PQ-4 containing VP 67% {poly[(2-ethyldimethylammonioethyl methacrylate ethyl sulfate)-*co*-(1-vinylpyrrolidone)], composition % weight: VP 67; DMAEMA 33, Mw ∼1,000,000 and charge density: 0.8}. This also confirms that the DMAEMA produces lower toxicity against QVI. Overall, all PQs showed reduced cytotoxicity compared with the PE-4 owning much higher charge density compared to PQs in view of their structures (Wang et al., 2015) and much lower against PEI 25k at the same dose. The cell viability rate was dropped to 12.6% when it is treated with PEI 25k, whereas PQ-1, PQ-2, PQ-3 and PQ-4 showed cell viability at 87.9%, 59.5%, 91.5% and 97.3% at the same dose of 50 μg/mL, respectively. Even at the dose of 100 μg/mL, PQ-1, PQ-3 and PQ-4 maintained viability at 81.5%, 92.3% and 97.7%, respectively, in contrast to only 2.5% with PEI 25k.

### 3.2. Delivery of PMO with PQs *in vitro*

**Figure 3.**
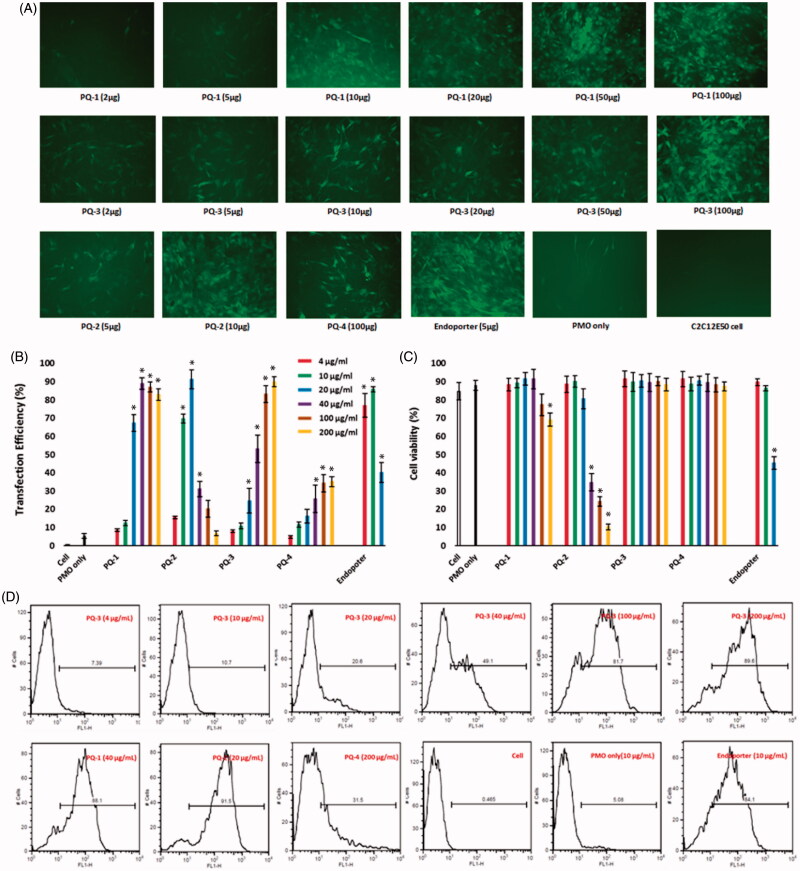
Delivery efficiency and toxicity of PMOE50/PQs complexes in C2C12E50 cell line determined by fluorescence microscope and FACS analysis. (A) Representative fluorescence images of PMO-induced exon-skipping in C2C12E50 cell line. The images were taken after three-day treatment, and original magnification: ×200. (B) Transfection efficiency of PMOs formulated with PQs (two-tailed *t*-test, *p ≤ .05 compared with PMO only). (C) Cell viability (two-tailed *t*-test, *p ≤ .05 compared with untreated cell as control). (D) Flow cytometry histogram (FL) of PMOE50/PQs complexes. In this test, PMOE50 (10 μg/mL) was formulated with PQs (4, 10, 20, 40, 100 and 200 μg/mL for each polymer), and Endoporter (4, 10, 20 μg/mL) formulated as control in 0.5 mL 10% FBS-DMEM medium, respectively. The results are presented as the mean ± SEM in triplicate.

The C2C12E50 myoblasts expressing a bifurcated green fluorescent protein (GFP) reporter by the insertion of the human dystrophin exon 50 (hDysE50) were used to evaluate the efficacy of PQs for the delivery of PMO (Sazani et al., 2001; Hu et al., 2010). The expression of GFP in the reporter cells relies on the targeted skip of exon 50 by AOs. The PMO sequence, PMOE50 (5′-AACTTCCTCTTTAACAGAAAAG CATAC-3′) with previously confirmed efficacy of targeted removal of hDysE50, was used (Hu et al., 2010; Wang et al., 2015). C2C12E50 GFP reporter cells were treated with a fixed amount (10 μg/mL) of PMOE50 in 0.5 mL 10% FBS-DMEM medium formulated with each polymer at six different doses (4, 10, 20, 40, 100 and 200 μg/mL). Transfection efficiency was examined by fluorescence-activated cell sorting (FACS) analysis (Figure 3). The results showed that almost all PQ polymers at 20 μg/mL improved GFP expression significantly compared with PMOE50 alone. The levels of GFP expression were PQs’ structure-dependent. For instance, the celling dose of Luviquat series (PQ-1/2/3) with maximum GFP expression is closely related to charge density: the higher charge density it is, the lower dose it needs to achieve highest GFP expression. As evidenced by the fact that over 90% GFP expression levels were achieved with PQ-1 (QVI 30, charge density 2.0) at the dose of 40 μg/mL; PQ-2 (QVI 50, and charge density 3.3) at the dose of 20 μg/mL; and PQ-3 (Vcap 50, QVI 10, charge density 0.5) at the dose of 200 μg/mL comparable to or higher than Endoporter-mediated delivery (10 μg/mL, effective dose *in vitro*, recommended by Gene Tools), whereas PQ-4 without QVI (DMAEMA 33, charge density 0.8, Mw: 1,000,000) produced only 35% expression rate at the dose of 200 μg/mL, despite it has the super Mw 1,000,000 and 0.8 charge density both higher than PQ-3 (Vcap 50, QVI 10, charge density 0.5, Mw 700,000). The GFP expression resulted from PQ-1/2/3-mediated PMO delivery was noted to be up to 15-fold higher when compared with PMO alone (less than 6%) under their optimum dosage. The exon-skipping efficiency is obviously dose-dependent and toxicity-related for PQs. Doses higher than those achieving the peak delivery rate inevitably resulted in lower viability and GFP expression, as seen with PQ-2 which produced peak GFP expression at the dose of 20 μg/mL, but then showed a sharp decline with higher doses because of its high toxicity resulted from its high charge density.

To assess the delivery potential of the PQs for PMO exon-skipping in muscle fibers, the PQs were further tested in C2C12E23 differentiating myotubes (Hu et al., 2010). The C2C12E23 reporter construct uses a muscle creatine kinase (MCK) promoter to drive the expression of GFP, thus allowing us to evaluate the exon-skipping of antisense oligonucleotides more specifically in differentiating or differentiated cells. Cells reaching around 70% confluence were switched to differentiation media for 2 days and then treated with PQs formulated with PMOE23. We examined the PQs at their optimal dosage based on the results from PMOE50 delivery: the PQ-1/3/4 (100 μg/mL), PQ-2 (20 μg/mL) and Endoporter (10 μg/mL as control) formulated with PMOE23 (10 μg/mL). After six-day delivery, the results showed a similar trend as obtained in C2C12E50 cells with PQ-1, PQ-2 and PQ-3 achieving higher GFP expression comparable to or higher than Endoporter-formulated PMO delivery as illustrated in Figure S1 (Supplementary Materials) by fluorescence images and RT-PCR detection of skipped exon 23. The levels of exon 23 skipping were 64.8%, 57.9%, 61.5%, 45.7%, 54.8% and 26.8% for PQ-1, PQ-2, PQ-3, PQ-4, Endoporter-formulated PMO and PMO only, respectively. The performances indicate the importance of the molecular size, the chemical structure and positive charge distribution in vector microstructure for PMO delivery applied to myoblasts and myotubes, and those could contribute to both delivery efficiency and toxicity of being as delivery vehicle.

### 3.3. Delivery of PMO with PQs *in vivo*

#### 3.3.1. Local delivery

We next evaluated the effect of the PQs for PMO delivery *in vivo* by i.m. injection. PMOE23 targeting mouse dystrophin exon 23 was injected to each TA muscle of *mdx* mice aged 4–5 weeks. The mouse contains a nonsense mutation in the exon 23, preventing the production of the dystrophin protein. Targeted removal of the mutated exon 23 can restore the reading frame of dystrophin transcripts and thus the expression of the dystrophin protein. Based on the delivery performance of PQs *in vitro*, we chose the dose of 20 μg as effective and safe dosage, to premix with 2 μg of PMOE23 in 40 μL saline. The treated TA muscles were harvested two weeks later.

**Figure 4.**
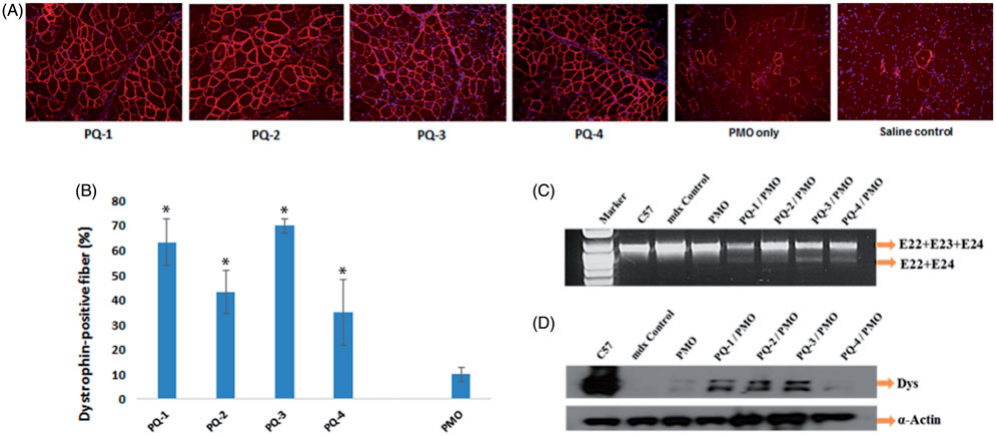
Restoration of dystrophin in TA muscles of *mdx* mice (aged 4–5 weeks) 2 weeks after i.m. injection. [The samples were from muscles treated with PQs (20 μg) and PMOE23 (2 μg) in 40 μL saline, PMOE23 only treated as controls]. (A) Dystrophin was detected by immunohistochemistry with rabbit polyclonal antibody P7 against dystrophin. Blue nuclear staining with DAPI, and original magnification: ×100. (B) The percentage of dystrophin-positive fibers in muscles treated with PQs formulated with PMOE23 (*n* = 5, two-tailed *t*-test, *p ≤ .05 compared with PMO alone). (C) Detection of exon 23 skipping by RT-PCR. Total RNA of 100 ng from each sample was used for amplification of dystrophin mRNA from exon 20 to exon 26. The upper bands (1093 bp, indicated by E22 + E23 + E24) correspond to the normal mRNA, and the lower bands (880 bp, indicated by E22 + E24) correspond to the mRNA with exon E23 skipped. (D) Western blots demonstrate the expression of dystrophin protein. Dys, dystrophin detected with monoclonal antibody Dys 1. α-Actin was used as loading control.

Immunohistochemistry showed that the TA muscles treated with PQ-formulated PMOE23 dramatically increased the numbers of the dystrophin-positive fibers, and the percentage was reached up to 64%, 42%, 70% and 33% (in one cross-section of the TA muscle) for PQ-1-, PQ-2-, PQ-3- and PQ-4-formulated PMO, respectively. Especially, the PQ-1/3 was more effective showing six/seven-fold increase over PMO only, which produced maximum of 11% positive fibers. The levels of exon-skipping and corresponding dystrophin expression were also quantitatively determined by RT-PCR and Western blot. PQ-1-, PQ-2-, PQ-3- and PQ-4-formulated PMO and PMO only achieved the levels of exon-skipping at 37.7%, 25.5%, 42.8%, 31.6% and 8.7%, respectively. Consistently, dystrophin protein expression levels were found to be 33.5%, 47.1%, 52.7%, 15.7% and 11.5% (related to the level of the protein in TA of C57 mice as 100%) for PQ-1-, PQ-2-, PQ-3- and PQ-4-formulated PMO and PMO only, respectively (Figure 4). These results correlated well with the data in muscle cell lines *in vitro*, indicating the importance of the composition and charge density within the vector molecule to improve the delivery efficiency of therapeutic agent in both *in vitro* and *in vivo*. Taken together, these results demonstrated that: (1) PQ-1/2/3 with cationic QVI performs better for PMO delivery than PQ-4 with cationic DMAEMA. This is probably due to the strong binding affinity between the PQ and PMO *via* hydrophobic interaction and additional ᴫ–ᴫ stacking, enabling PQs with QVI to form a rigid complex with PMO, resulting in higher transfection efficiency; (2) the moderate or low positive charge density of vector molecule could be sufficient for efficient delivery of uncharged PMO, such as the PQ-1 (charge density: 2.0) and PQ-3 (charge density: 0.5) showed better behavior than PQ-2 (charge density: 3.0); (3) a much higher transfection efficiency of PQ-3 (Mw: 700,000; Vcap 50, QVI 10, charge density: 0.5) than that of PQ-1 (Mw:100,000; QVI 30, charge density: 2.0) suggests that the larger molecular size and more hydrophobicity also play important role, probably providing higher freedom for PMO binding, compensating for its lower positive charge. And the surface charged PQ-3/PMO complex might make it stable enough in biological condition compared with naked PMO (Hoshino et al., 2012; Nakamoto et al., 2014). As we know, *in vivo* delivery requires not only the ability of a vector to introduce therapeutic agent into the cells but also an efficient delivery of the agent to the vicinity of the cells/tissues. After administration into the blood circulation, the vector/therapeutic agent complex interacts with various cells and biomacromolecules due to charge and supramolecular reaction (Barron et al., 1998; Sakurai et al., 2001). These results further highlight the complexity of the interaction between polymer-based vector and their delivery cargos.

#### 3.3.2. Systemic delivery

**Figure 5.**
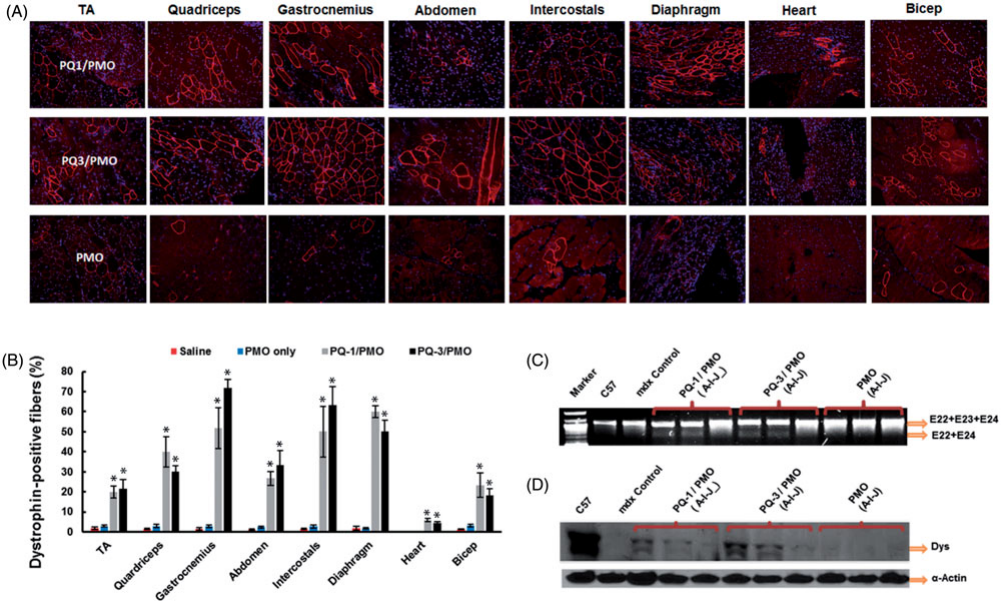
Restoration of dystrophin expression after two-week systemic delivery of PMO with PQs in *mdx* mice (aged 4–5 weeks). Each mouse was injected with 1 mg PMOE23 with and without PQs (0.5 mg). (A) Dystrophin was detected by immunohistochemistry with rabbit polyclonal antibody P7 against dystrophin. Blue nuclear staining with DAPI, and original magnification: ×100. (B) Percentage of dystrophin-positive fibers in different muscle tissues (mean ± SEM, *n* = 5, two-tailed *t*-test, *p ≤ .05 compared with 1 mg PMO only). (C) Detection of exon 23 skipping by reverse transcription polymerase chain reaction. Total RNA of 100 ng from each sample was used for amplification of dystrophin mRNA from exon 20 to exon 26. The upper bands correspond to the normal mRNA, and the lower bands correspond to the truncated mRNA with exon E23 skipped. (D) Western blots demonstrate the expression of dystrophin protein from treated *mdx* mice in comparison with C57BL/6 and untreated *mdx* mice (20 μg of total protein was loaded for PQ-formulated PMO, PMO-treated mice, WT C57 and control *mdx*. A: TA; I: Diaphragm; J: Heart). Dystrophin detected with monoclonal antibody Dys 1. α-Actin was used as the loading control.

DMD affects body-wide muscles including cardiac muscle, and systemic treatment is therefore indispensable. Based on the results *in vitro* and *in vivo* locally, we selected the two most effective polymers (PQ-1 and PQ-3) to evaluate their effects for PMO systemic delivery by intravenous (i.v.) injection at the dose of 0.5 mg formulated with 1 mg PMOE23 (Figure 5). The control PMOE23 (1 mg) alone induced dystrophin expression in less than 3% of muscle fibers in all skeletal muscles and no detectable dystrophin in cardiac muscle two weeks after injection. PMOE23 formulated with both PQs produced dystrophin positive fibers over 20% in all skeletal muscles except for the bicep (18% with PQ-3). Over 50% positive fibers observed in gastrocnemius, intercostals and diaphragm with both PQs, and over 60% and 70% in gastrocnemius and intercostals with PQ-3, respectively. Importantly, immunohistochemistry demonstrated membrane-localized dystrophin in about 7% and 5% of cardiac muscle fibers in some areas of the heart with the single dose PQ-1/PMO and PQ-3/PMO, respectively. In contrast, only occasional one or two positive fibers were observed in cardiac tissue in the mice treated with PMO alone. The levels of dystrophin restoration are consistent with the significant reduction of serum creatine kinase levels (Figure S2, Supplementary Materials). Dystrophin induction even at low levels in cardiac muscle could be highly beneficial to the patients, because cardiomyopathies due to lack of dystrophin expression are currently the leading cause of death among DMD patients (Cirak et al., 2011; Mendell et al., 2013). In addition, restoration of dystrophin in skeletal muscles alone may exacerbate the failure of heart function as a result of increased workload.

Consistent with the immunohistochemistry, RT-PCR results showed that TA and diaphragm muscles with PQ-formulated PMO-treated mice contained spliced form of dystrophin mRNA with exon 23 skipped as being about 20–30% of the total product. Expression levels of dystrophin protein in TA and diaphragm muscles measured by Western blots for PQ-3-formulated PMO-treated mice were around 20–30% of the levels of the normal control muscle obtained, respectively. It is difficult to detect in the heart caused by low level (only 5–7%) of dystrophin positive fibers observed. PQ-1-formulated PMO-treated ones were slightly lower as compared to PQ-3-formulated PMO administration, which is in line with the exon-skipping outcome of local delivery.

No signs of abnormal behavior or change in body weight and overall condition were observed during systemic treatment with PQ-formulated PMO, and no pathologic changes of the liver, kidney and lung of the treated mice were detected by H&E staining and levels of serum enzymes (Figure S2, Supplementary Materials). These results solidify the PQs as the potential vectors for antisense PMO delivery to increase exon-skipping efficiency especially for the treatment of muscular dystrophies.

### 3.4. Interaction between PQ and PMO

**Figure 6.**
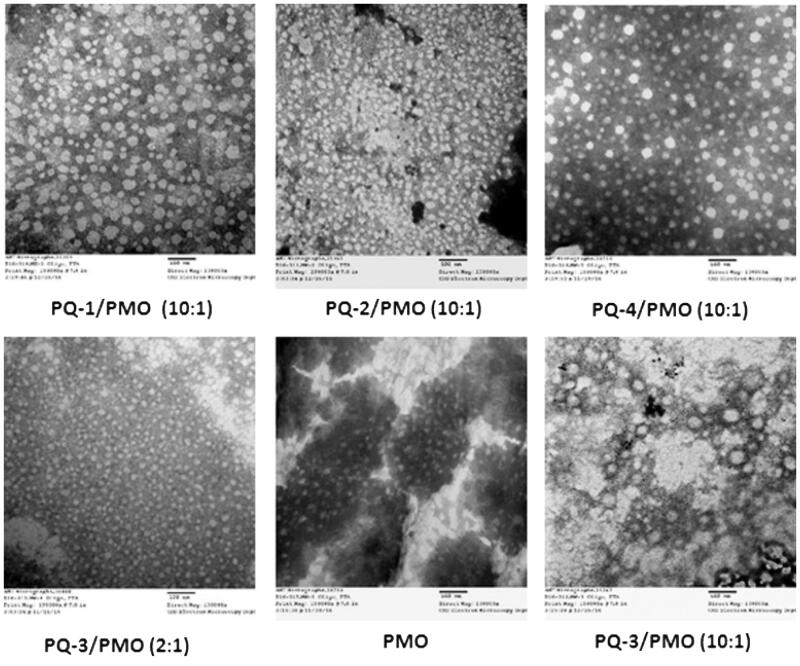
Negatively stained TEM images of PQs complex with PMO (the polymer/PMO polyplex solution containing 1 μg of PMO at a weight ratio of 10/1 or 2/1 (PQ/PMO) in 100 μL 0.9% saline. Direct magnification: ×130,000, scale bar: 100 nm).

The affinity between polymer and oligonucleotide is an important parameter for their efficient delivery into cells or tissues. To understand how the PQs improve the delivery performance of oligonucleotide PMO, we herein examined PQs/PMO polyplex at the weight ratio of 10 in 0.9% saline solution under TEM based on the *in vitro* and *in vivo* results, and PQ-3/PMO at the ratio of 2 also was assessed. As illustrated in Figure 6, the polymer PQ-1/PMO complex formed particles sized varying from 10 to 70 nm likely because of the PQ-1’s higher charge density 2.0 (pH = 7), leading to some degree of particle aggregation; the PQ-2/PMO complex under same weight ratio produced condensed particles sized below 30 nm probably due to the PQ-2’s more higher charge density 3.3 (pH = 7), relative higher hydrophilicity (QVI 50%) and smaller molecular size (∼80,000) relative to PQ-1 (QVI 30%, Mw: 100,000); the PQ-3/PMO aggregated to form big particles resulted from its great Mw (∼700,000), higher lipophilicity (QVI 10%, Vcap 50%) and lower charge density 0.5 (pH = 7) as compared to PQ-1 or PQ-2. while, at the low weight ratio 2 of PQ-3/PMO, smaller and regular particles sized down to below 20 nm observed, that is likely less aggregation. PQ-4/PMO polyplex-formed particle with the size similar to PQ-1/PMO polyplex, likely caused by its huge molecular size (∼1,000,000) compared with PQ-1 (∼100,000) with higher flexibility to condense PMO, compensating for its lower charged density 0.8 (pH = 7), whereas the PMO oligonucleotides alone formed particles with the size below 50 nm, probably a result of hydrophobic interaction and hydrogen bond among PMO molecules. The mechanisms of interaction between PMO and PQs molecules are not clear, but the chemical nature of PMO likely endows a hydrophobic interaction with the PQ polymers and possible hydrogen bond interaction among them. On the other hand, the moderate positive charges of polymer as delivery vehicle for uncharged PMO still play the role, though it is not such key as in delivery for negatively charged oligonucleotides. Because the positive surface charges of the polyplex may stabilize polymer/PMO polyplex for a longer period than PMO alone under biological environment. This may lead to a higher serum level tolerance and improvement in the uptake of PMO through the vasculature and cell membrane, and thus more effective delivery of PMO into the muscles.

## 4. Conclusions

In this study, the cationic PQs have been evaluated for the first time as vector for antisense PMO delivery *in vitro* and in dystrophic *mdx* mice. The results show that Luviquat series, especially PQ-1 and PQ-3, improve the delivery efficiency of PMO to the levels comparable to Endoporter-mediated PMO delivery *in vitro*. The significant enhancement of PMO delivery is also demonstrated *in vivo*, up to seven-fold with PQ-3 when compared with PMO only. No obvious toxicity was observed in local and systemic delivery at the tested dosage. These data suggest that optimization of PQs in molecular size, component and charge density can achieve enhanced PMO-mediated exon-skipping with potential to realize therapeutic value. Further study is required to establish toxicity and long-term efficacy by repeated administration *in vivo*.

## Supplementary Material

IDRD_Mingxing_et_al_Supplemental_Content.docx
