# Supplementary material for: Polyquaternium-mediated delivery of morpholino oligonucleotides for exon-skipping in vitro and in mdx mice
Source: Drug Deliv. 2017 Jun 20;24(1):952–61. doi: 10.1080/10717544.2017.1337827 (PMC8241187; doi:10.1080/10717544.2017.1337827)
Supplement: IDRD_Mingxing_et_al_Supplemental_Content.docx [file IDRD_A_1337827_SM5877.docx]

**Supplementary Materials**

**Polyquaternium-mediated Delivery of Morpholino Oligonucleotides for Exon-skipping *in vitro* and in *mdx* Mice**

Mingxing Wang*, Bo Wu, Sapana N Shah, Peijuan Lu, Qilong Lu

McColl^-^Lockwood Laboratory for Muscular Dystrophy Research, Cannon Research Center,

Carolinas Medical Center, 1000 Blythe Blvd. Charlotte, NC 28203, USA

*Tel.: 1-704-355-5588; Fax: 1-704-355-1679; Email: [mingxing.wang@carolinashealthcare.org](mailto:mingxing.wang@carolinashealthcare.org)*


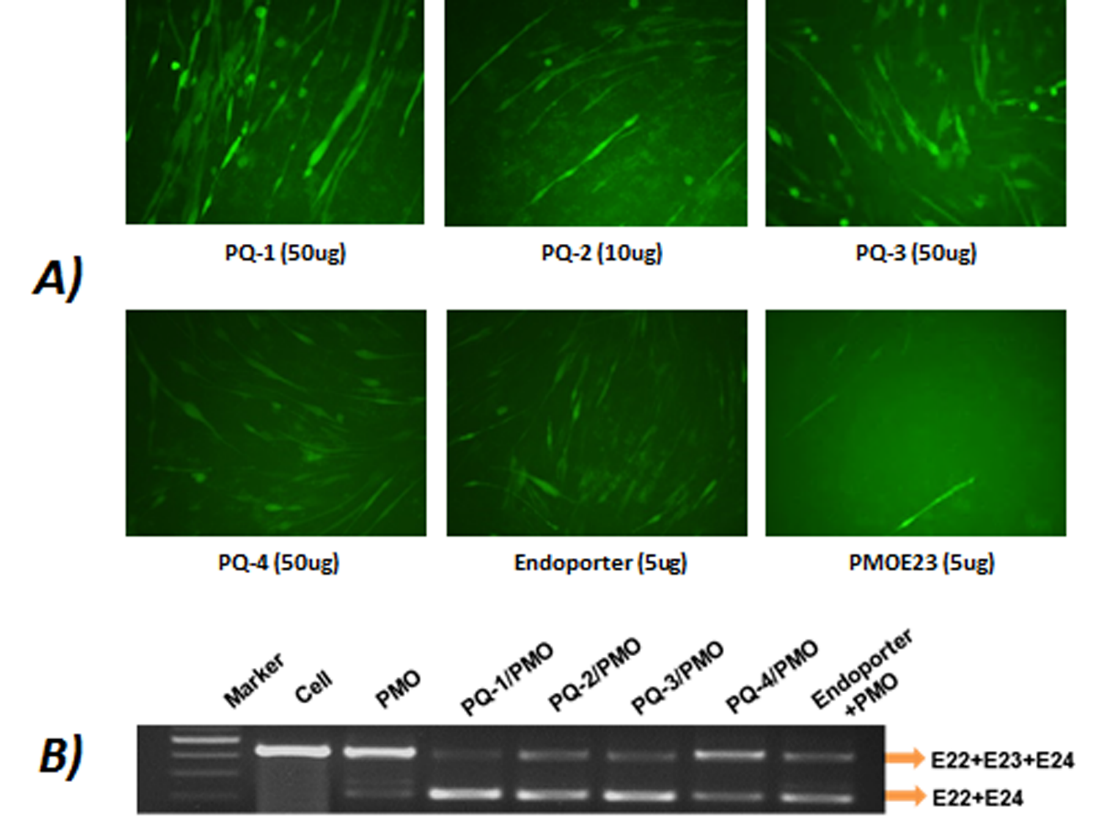


**Figure S1**. GFP expression induced by PMOE23 (10 µg/mL) formulated with PQs in C2C12E23 cells [PQ-1/3/4 (100 µg/mL), PQ-2 (20 µg/mL), Endoporter (10 µg/mL) in 0.5 mL 10% FBS-DMEM after 6-day treatment]. **A**) Fluorescence detection for GFP expression, and original magnification: x100. **B**) RT-PCR of exon 23 skipping. The upper bands (424 bp, indicated by E22+E23+E24) correspond to the normal.


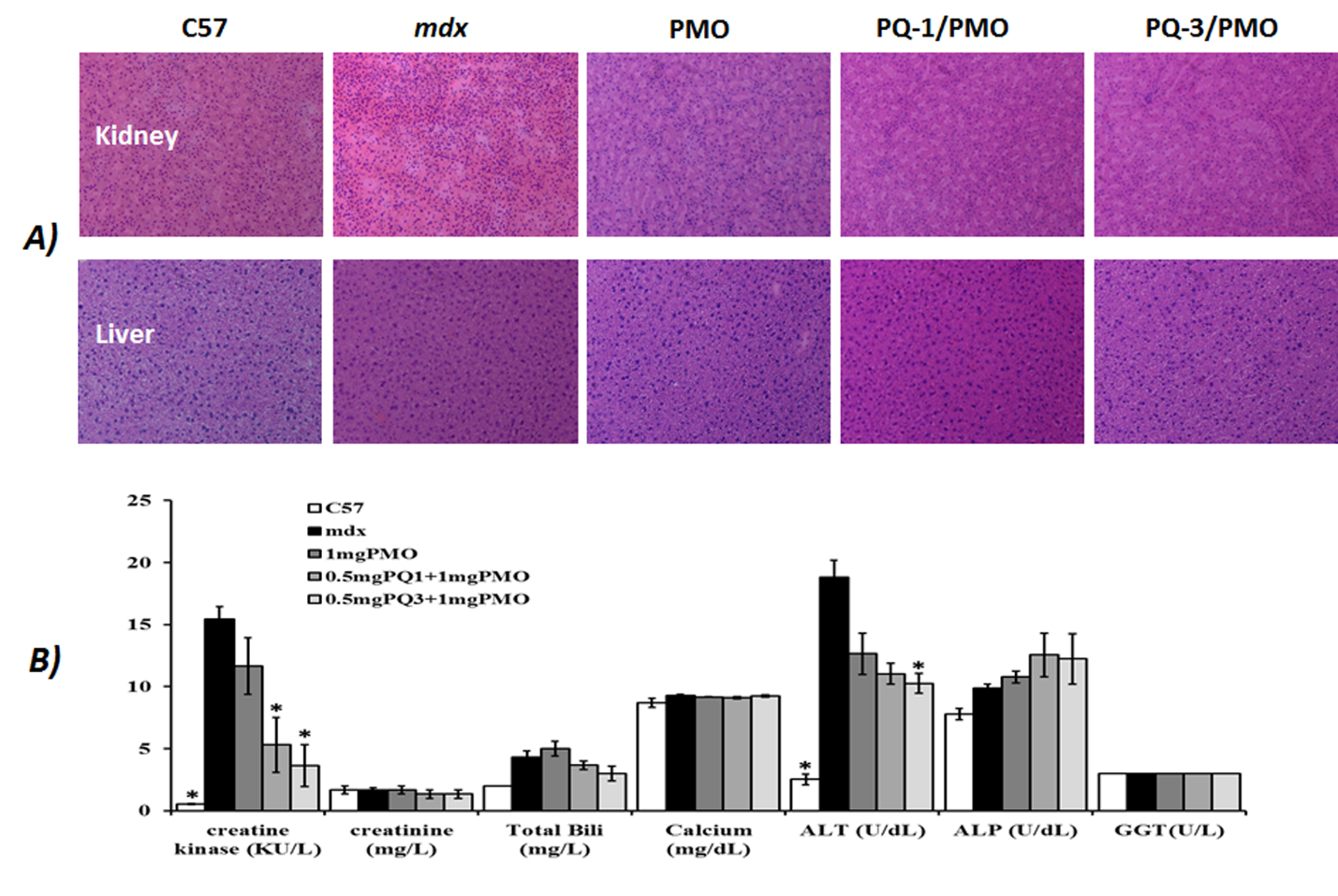


**Figure S2.** Examination of pathology and serum tested after 2-week systemic delivery of PMO with PQs in *mdx* mice (aged 4-5 weeks). Each mouse was injected with 1 mg PMOE23 with and without PQs (0.5 mg). The normal C57BL6 mice (C57) as positive control, untreated *mdx* mice as negative controls. **A**) Hematoxylin and Eosin (H & E) staining of liver and kidney tissues. Original magnification: x200. **B**) The levels of serum enzymes, creatine kinase (KU/L), creatinine (mg/L), total bilirubin (mg/L), direct bilirubin (mg/dL), alanine transaminase (ALT, U/dL), alkaline phosphatase (ALP, U/dL), and ɤ-glutamyltransferase (GGT, U/L) (mean ± SEM, n = 5; Two-tailed t-test, **p* ≤ 0.05 compared with untreated *mdx* mice).
